# Small Cell Carcinoma of Ovary, Hypercalcemic Type (Malignant Rhabdoid Tumor of Ovary) with Loss of SMARCA4 (BRG1) Expression: Report of Two Cases

**DOI:** 10.5146/tjpath.2020.01477

**Published:** 2020-09-15

**Authors:** Ayushi Sahay, Katha Kante, Santosh Menon, Jaya Ghosh, Rajendra A. Kerkar, Kedar K. Deodhar

**Affiliations:** Departments of Pathology, Tata Memorial Centre, Homi Bhabha National Institute, Mumbai, India; Departments of Medical Oncology, Tata Memorial Centre, Homi Bhabha National Institute, Mumbai, India; Departments of Surgical Oncology, Tata Memorial Centre, Homi Bhabha National Institute, Mumbai, India

**Keywords:** Small cell carcinoma ovary, Rhabdoid tumor of ovary, BRG1, Ovarian tumor

## Abstract

Small cell carcinoma of the ovary, hypercalcemic type (SCCOHT) / malignant rhabdoid tumor of the ovary (MRTO) is a rare tumor affecting young women. It is frequently misdiagnosed due to overlapping morphological and immunohistochemical features with many other ovarian tumors. The prognosis of the tumors is very poor; hence an accurate diagnosis is of utmost importance. Recently, the loss of BRG1 protein by immunohistochemistry has been shown to be a useful diagnostic marker. We present here two cases of SSCOHT/MRTO, in young women 22 and 32 years of age, where several differential diagnoses were considered on morphology and immunohistochemistry but were confirmed as SCCOHT/MRTO by the demonstration of loss of BRG1. As the prognosis of SCCOHT is very dismal, and accurate diagnosis is of necessity, we recommend the inclusion of BRG1 immunohistochemistry in the diagnostic armamentarium of poorly differentiated ovarian tumors, particularly in young adults.

## INTRODUCTION

Ovarian small cell carcinoma of hypercalcemic type (SCCOHT) is a rare and highly aggressive tumor of young adults with uncertain histogenesis and very poor prognosis (1). Malignant rhabdoid tumors (MRT) have been rarely reported in the ovaries. Recently, near-simultaneous genomic studies demonstrated that SCCOHTs show inactivating mutations in SMARCA4, accompanied by loss of expression of its protein product BRG1, the same mutation that has been described in a proportion of atypical teratoid/rhabdoid tumors (ATRT) of the brain as well as extracranial MRT (2–4). In addition, SCCOHTs show histological similarity with MRT, with the presence of small undifferentiated round cells, as well as large rhabdoid looking cells in up to 40% of the cases (the so-called “large cell variant”). Based on this histologic and genetic similarity, it has been proposed to rename SCCOHT of the ovary as MRT of the ovary (MRTO) (5). In most prior studies, the primary diagnosis of SSCOHT was based on morphology, which can mimic a large variety of other ovarian tumors, and testing for SMARCA4 or BRG1 was done only retrospectively in morphologically diagnosed cases (5–7). There are very few cases of SCCOHT reported prospectively based on loss of BRG1 (8). We present here two cases of MRTO, which posed a diagnostic conundrum, and were diagnosed by demonstration of loss of BRG1 on immunohistochemistry.

## CASE REPORT

### Case 1

A 22-year-old unmarried female presented with lower abdominal pain for two months associated with fever. On per abdomen examination there was minimal hypogastric tenderness and a large mobile pelvic mass in the left iliac fossa extending to the umbilical region. Ultrasonography revealed a large 18x17 cm cystic to solid mass lesion in the lower abdominal and pelvic cavity, without calcification, with ascites and minimal bilateral pleural effusion. On investigation, her serum CA125 was 841 U/ml (range 0-35 U/ml), LDH was 361 U/L (range 100-190U/L), AFP was 1.3 ng/ml (0.89-8.78 ng/mL), CEA was 2.1 ng/ml (range 0-3 ng/ml), CA19-9 was 2 U/ml (range 0-37 U/ml), and Serum B-HCG was 0.79 mIU/ml (range 0-5 mIU/ml). Clinically germ cell tumor was strongly suspected. She underwent an explorative laparotomy with left salphingoophorectomy with omentectomy. In the intraoperative frozen section, a poorly differentiated malignant tumor with the possibility of dysgerminoma was suggested.

**Figure 1:**
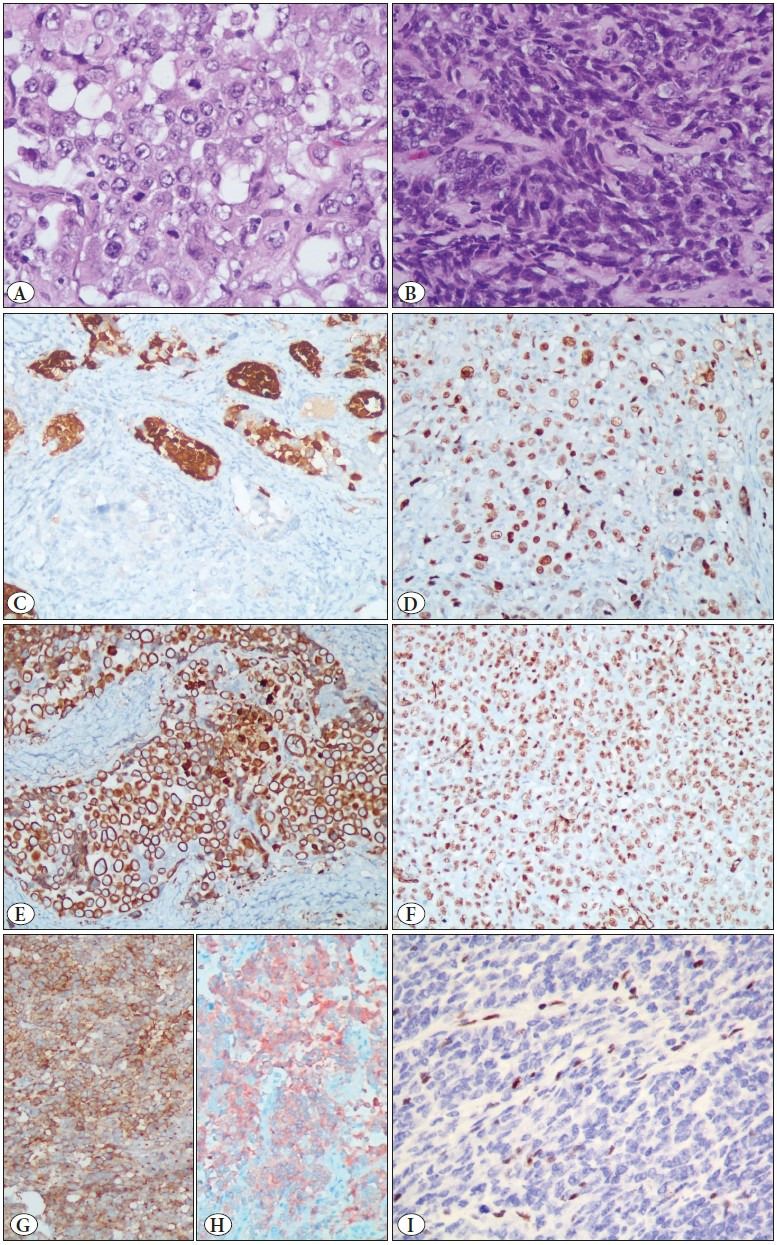
Photomicrographs of case 1 showing **A)** Sheets of large rhabdoid cells with abundant eosinophilic cytoplasm, vesicular round nuclei with prominent nucleoli, and frequent mitosis (H&E; x 400). **B)** Sheets of malignant round cells with scant cytoplasm and hyperchromatic nuclei (H&E; x 400). **C)** Strong cytoplasmic and nuclear immunopositivity for calretinin (IHC; x200). **D)** Strong nuclear immunopositivity for p53 (mutant type) (IHC; x200). **E)** Diffuse cytoplasmic immunopositivity for AE1/AE3 (IHC; x200). **F)** Diffuse nuclear immunopositivity for WT1 (IHC; x100). **G)** Patchy membranous and dot-like immunopositivity for MIC- 2 (CD99) (IHC; x200). **H)** Diffuse moderate immunopositivity for synaptophysin (IHC; x200). **I)** Immunostaining for BRG1 demonstrating loss of BRG1 in the tumor cell nuclei, while normal endothelial cell nuclei can be seen as internal control (IHC; x400).

On gross examination, the left ovarian mass measured 17x16x9 cm with a bosselated external surface and capsular breach. The cut surface was solid-cystic, with cysts containing hemorrhagic fluid, and grey-white solid areas with hemorrhage and necrosis. The omentum showed a metastatic tumor deposit. On microscopic examination, the tumor was composed of sheets of malignant small round cells juxtaposed with areas showing islands and nests of large cells with abundant eosinophilic cytoplasm, and large vesicular nuclei with prominent nucleoli (Figure 1). At places, mucinous degeneration with attempt at follicle formation was noted. Focal areas of hyalinization with tumor cells arranged in cords was seen. Frequent mitoses and lymphovascular emboli were noted.

On morphology, differentials of a malignant sex cord-stromal tumor, poorly differentiated adenocarcinoma, neuroendocrine tumor, and germ cell tumor were entertained. Accordingly, immunohistochemistry was performed, the results of which are summarized in Table 1 (Figure 1).

**Table 1:** Summary of immunohistochemical findings.

	**Case 1**		**Case 2**	
	**Positive**	**Negative**	**Positive**	**Negative**
**Markers for Epithelial lineage**	AE1/AE3, EMA, P53 (mutant type), WT1	CK7, CK20	AE1/AE3, EMA, WT1, CK19	P53 (wild type), CK7, TTF1
**Markers for Sex Cord Stromal lineage**	Calretinin, Mic-2 (CD99)	Inhibin	Mic2 (CD99),	Calretinin, Inhibin
**Markers for Neuroendocrine Lineage**	CD56, Synaptophysin	-	CD56, Synaptophysin	Chromogranin
**Markers for Germ Cell Tumor Lineage**	-	CD30, Glypican3, KIT (CD117), Oct3/4	-	AFP, PLAP, KIT (CD117)
**Miscellaneous Markers**	CD10 focal	Desmin	CD10	Bcl2, S100, TLE1
**INI1**	Retained		Retained	
**BRG1**	Loss		Loss	

**Figure 2:**
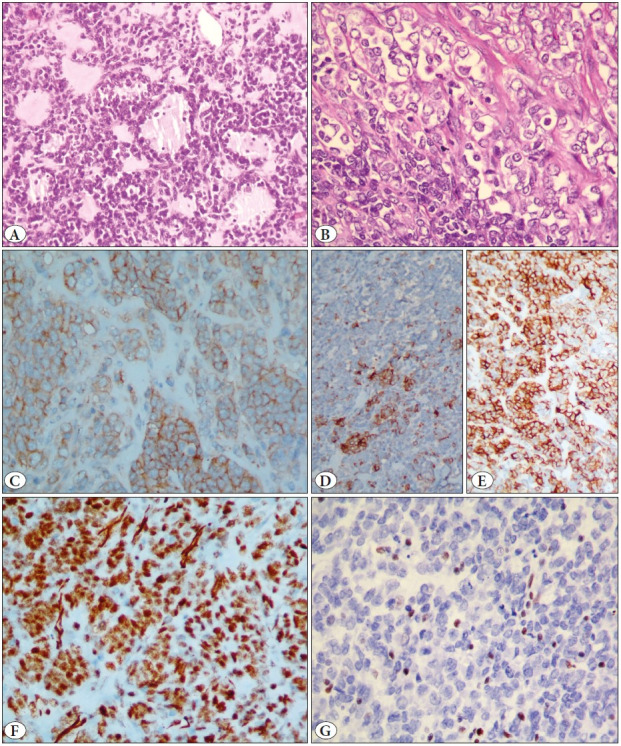
Photomicrographs of case 2 showing **A)** Round cells with scant cytoplasm and hyperchromatic nuclei, showing mucinous degeneration with formation of follicle-like structures (H&E; x 200). **B)** At other places, the tumor cells showed more abundant cytoplasm, with vesicular nuclei and distinct to prominent nucleoli (H&E; x400). **C)** Moderate membranous immunopositivity for MIC-2 (IHC; x400). **D)** Patchy cytoplasmic positivity for AE1/AE3 (IHC; x100). **E)** Diffuse cytoplasmic immunopositivity for CD56 (IHC; x200). **F)** Diffuse nuclear immunopositivity for WT1 (IHC; x400). **G)** Loss of BRG1 protein in tumor cells with immunopositive internal control (Blood vessels and inflammatory cells) (IHC; x400).

It can be seen from Table 1 that except for germ cell tumor (for which all the markers were negative), the tumor was polyphenotypic and showed a significantly overlapping immunohistochemical profile for epithelial, neuroendocrine as well as sex cord-stromal lineage. This, taken together with the young age of the patient, was suggestive of small cell carcinoma of the ovary, hypercalcemic type (SSCOHT), now called malignant rhabdoid tumor of the ovary. The patient’s preoperative serum calcium levels were not available, and postoperative levels were found to be within a normal range. We performed immunohistochemistry for BRG1 protein which showed loss of the protein in the tumor cells, confirming the diagnosis (Figure 2).

Following the diagnosis, the patient was started on cisplatin and etoposide based chemotherapy. However after 3 cycles, a follow-up CT scan showed disease progression with an increase in the size of the pelvic mass, retroperitoneal nodes, and the hepatic and spleen metastatic deposits. She was started on palliative chemotherapy with weekly paclitaxel and supportive care. However, there was no response and she succumbed to the disease within four months of diagnosis.

### Case 2

A 32-year-old female, presented with an abdominal mass and underwent explorative laparotomy for a right ovarian cyst. Preoperatively, CA125 was 36 U/ml (range 0-35 U/ml), CA19-9 was 32 U/ml (range 0-37 U/ml), while CEA, AFP, inhibin, and b-HCG were within normal limits. Intraoperatively, the right ovary showed a cystic mass measuring 25 cm in the largest dimension, and multiple 1-3 cm omental, peritoneal, bladder, mesenteric and pouch of Douglas deposits. An intraoperative frozen section confirmed malignancy and hence total abdominal hysterectomy with bilateral salphingo-oophorectomy, omentectomy and peritoneal deposit removal was carried out. Subsequently, the patient presented to our hospital for further management and we received the slides and block of the ovarian tumor for review. On microscopic examination, both the ovaries and all metastatic deposits showed similar tumor morphology, composed of large rhabdoid cells interspersed with sheets of malignant round cell-like areas. Scattered follicle-like structures were identified (Figure 2). A spindle morphology was seen. Frequent mitosis was observed. Foci of myxoid background were noted. A differential of poorly differentiated sex cord-stromal tumor, high-grade neuroendocrine tumor, and peripheral neuroectodermal tumor was entertained. Immunohistochemical findings are summarized in Table 1 (Figure 2).

The tumor, similar to the first case, showed polyphenotypic expression of markers of epithelial, sex cord-stromal and neuroendocrine lineage, and thus small cell carcinoma of the ovary (malignant rhabdoid tumor) was suspected. Immunohistochemistry for BRG1 showed a loss of BRG1 protein expression, confirming the diagnosis (Figure 2).

Pre-surgery, serum calcium levels were very mildly elevated (11.4 mg/dl). Within three months of surgery, the patient developed umbilical nodules and a CECT scan showed multiple lesions in the hepatorenal space, left hypochondrium, pelvis, anterior abdominal wall, and enlarged nodes. On examination, multiple palpable nodules were felt above the vault compressing the rectum. The patient was started on etoposide and cisplatinum based chemotherapy. There was a clinical response in the first 2 weeks. However, by the end of the third cycle, there was a rapid increase in pain and the size of the umbilical nodule. The patient was sent for palliative care and was subsequently lost to follow up.

## DISCUSSION

SCCOHT was first described in 1979 by Robert Scully in his first fascicle on ovarian tumors (9). Only about 350 cases have been reported, with nearly half of them in a single large study by Young et al. (1). Although the tumor is exceedingly rare, it is the most common undifferentiated ovarian malignancy in women < 40 years of age. It is seen mainly in the second and third decade (mean age 24 years). In the large series by Young et al., preoperative hypercalcemia was seen in nearly two thirds (62%) of the women. The tumor is almost always unilateral and shows extra ovarian spread in nearly half the cases (1,10,11). There are reports suggesting possible familial inheritance. On gross examination, the tumors are large (mean diameter 15 cm) and predominantly solid. In both our cases, the patients were young and showed large ovarian masses with an extra ovarian spread. Preoperative calcium levels were not measured in one case, while in the other, the level was mildly elevated.

Microscopically, the tumor cells are arranged in diffuse sheets, nests, cords, and trabeculae, with interspersed variable follicle like spaces containing eosinophilic fluid in nearly 80% of the cases. As per the name, the tumor is mainly composed of small round cells with hyperchromatic nuclei and brisk mitosis, but nearly half the cases described also show a variable population of cells with abundant eosinophilic cytoplasm, sometimes containing hyaline globules, with vesicular nuclei and prominent nucleoli (1,10,11). Some authors described cases with a predominant large cell population as the “large cell variant” of SCCOHT- an oxymoron (5). Other microscopic morphological variations described include the presence of a minor component of the mucinous epithelium, sometimes forming glands or cysts, signet cells, spindle cells and stromal edema, myxoid change or hyalinization. Lymphovascular emboli are commonly seen (11). Because of these variability in features, the tumor can be confused with many primary and secondary neoplasms of the ovary such as sex cord-stromal tumor, including juvenile and adult granulosa cell tumor, germ cell tumor, endometrial stromal sarcoma, primitive neuroectodermal tumor (PNET), neuroblastoma, intra-abdominal desmoplastic small round cell tumor, undifferentiated carcinoma, lymphoma, malignant melanoma, and metastatic small cell neuroendocrine carcinoma. In both our cases, intermixed areas of small cells and large rhabdoid looking cells were encountered, with interspersed follicles in a variably myxoid and hyalinized stroma.

The same capriciousness of morphology is also reflected in the variable and non-specific immunohistochemical profile of SCCOHT. The majority of the tumors are reactive for p53, WT1, CD10 and one or more cytokeratins, about a third for EMA. Over half are positive for vimentin, and many for neuroendocrine markers, and calretinin. The tumors are negative for TTF1, desmin, and alpha-inhibin, and show retained expression of INI1 (1,11–13). Due to the morphological and immunohistochemical overlap, and its rarity, the diagnosis of SCCOHT remained challenging, even by expert pathologists, and many cases on review were usually diagnosed as another entity initially (5,6). Till recently, there was no specific confirmatory marker for SCCOHT. In 2013, Kupryjańczyk et al. noticed the clinical, histological and molecular similarities between SCCOHT, atypical teratoid/rhabdoid tumor (AT/RT) of the central nervous system, and malignant renal rhabdoid tumor (MRT). All these tumors occurred at a young age, showed rhabdoid large cells with admixed small cells and a polyphenotypic immunohistochemical profile, were genetically stable and had an exceedingly poor prognosis. Based on this observation, Kupryjańczyk et al. first tested two cases of SSCOHT for loss of INI-1 protein expression (the protein product of SMARCB1 gene), which was the known molecular diagnostic feature of MRT and AT/RT. When INI1 was retained in both the tumors, they went ahead and tested for loss of SMARCA4 immunohistochemical expression, which was the other rarer mutation described in a small percentage of AT/RT with retained INI1. INI-1 protein is a core subunit, while SMARCA4 protein is a catalytic ATPase subunit of the switching and sucrose non-fermenting (SWI/SNF) chromatin-remodeling complex critically involved in gene transcription. Both their cases showed loss of SMARCA4 on immunohistochemistry, and they confirmed the presence of SMARCA4 mutations in both the tumors by DNA sequencing (14). Subsequently, in 2014, three independent studies almost simultaneously carried out next-generation sequencing or whole-exome sequencing and demonstrated that SCCOHT show inactivating mutations in the SMARCA4 gene, accompanied by loss of protein expression of its product BRG1 (2–4). In one study, inactivating bi-allelic SMARCA4 mutations were seen in all 12/12 cases of SSCOHT (100%), accompanied by clear loss of protein expression of BRG1 in seven out of nine of these cases (2). Another study demonstrated germline or somatic mutations in familial cases of SSCOHT and showed loss of BRG1 immunohistochemistry in 38/40 familial and non-familial cases (4). The third study demonstrated loss of SMARCA4 protein in 14/17 SSCOHT and only 2/485 other primary ovarian tumors (0.4%). Both of the non-SSCOHT tumors with loss of BRG1 were clear cell carcinomas (CCC) (3). It was then that the Foulkes et al. claimed, putting two and two together, that based on the clinical, morphological, and now proved molecular similarity, SCCOHT are in fact MRT of the ovary, and should be renamed as such (5). Following these seminal observations, a large retrospective study showed loss of BRG1 with retained INI1 in 12/12 cases of SSCOHT and retained BRG1 expression (some with variable staining proportion and intensities) in 119 other tumors that can mimic SSCOHT morphologically. They concluded that immunohistochemical loss of BRG1 is a useful marker for SSCOHT, but advised caution in the interpretation of BRG1 on small biopsies due to the possibility of variability in staining (7). Conlon et al. found BRG1 loss in 16/17 (94%) SSCOHT and only 2/279 (0.7%) of other poorly differentiated ovarian tumors (both primary and metastatic). One of the non-SSCOHT cases which showed BRG1 loss was a CCC, while the other was a melanoma. Overall, they concluded that with a sensitivity of 94% and specificity of 99.3%, loss of BRG1 immunohistochemical expression is highly useful to distinguish SSCOHT from its many morphological mimics (6). Still, very few cases have been prospectively diagnosed based on BRG1 loss (8). The only other primary ovarian tumor which has rarely shown BRG1 loss in previous studies is CCC ovary, which is a tumor of older patients and can be differentiated from SSCOHT on morphology by the presence of clear cells, nuclear hobnailing, and absence of small round cells. Also, CCC does not show the polyphenotypic immunohistochemical expression typical of SSCOHT, but is, instead, positive for Napsin A. Among non-ovarian solid tumors, inactivating mutations of SMARCA4 have been rarely described in lung adenocarcinomas, and were associated with a poor outcome (2).

Although the mutation can be detected and confirmed by DNA sequencing methods, loss of protein expression of BRG1 by immunohistochemistry is a relatively inexpensive and more easily available technique. Due to the rarity of this tumor, treatment guidelines are not well defined. Thus, incorporation of BRG1 immunohistochemistry in the diagnostic armamentarium will possibly increase the number of accurately diagnosed cases, and may lead to the formulation of more effective management guidelines. Not only diagnostic, BRG1 loss carries implications for classification of these tumors, for genetic counseling of the patient’s family and in the future may be a candidate for epigenetic therapies (2,8,10). The prognosis is generally dismal, and despite multimodality treatment, median survival is much less than two years (10). Thus, a precise diagnosis is important, so aggressive multimodality therapy can be instituted. In both of our cases, a variety of differential diagnoses were considered on morphology. The immunohistochemical results were highly confusing with markers positive for epithelial, sex-cord stromal as well as neuroendocrine lineage (Table 1). Even though SCCOHT was suspected based on the presence of polyphenotypic ovarian tumors in young females, the diagnosis was conclusively established by demonstration of loss of BRG1, and chemotherapy was immediately initiated. However, in keeping with the known prognosis of this tumor, both the patients showed rapid disease progression.

To conclude, BRG1 is a novel immunohistochemical diagnostic marker which can clinch the diagnosis in suspected cases of SCCOHT, and should be included in the immunohistochemical evaluation of polyphenotypic ovarian tumors in young females. Accurate diagnosis of this rare tumor is of paramount importance since the tumor is resistant to all forms of therapy and has a dismal prognosis.

## Conflict of Interest

The authors declare no conflict of interest.

## FUNDING

This research received no specific grant from any funding agency in the public, commercial, or not-for-profit sectors.
